# An Evaluation of Drug-Drug Interaction Alerts Produced by Clinical Decision Support Systems in a Tertiary Hospital

**DOI:** 10.7759/cureus.43141

**Published:** 2023-08-08

**Authors:** Abdullah Alanazi, Wejdan alalawi, Bakheet Aldosari

**Affiliations:** 1 Health Informatics, King Saud Bin Abdulaziz University for Health Sciences, Riyadh, SAU; 2 Research, King Abdullah International Medical Research Center, Riyadh, SAU; 3 Nursing, Prince Mohammed Bin Abdulaziz Hospital, Medina, SAU

**Keywords:** computerized provider order entry (cpoe), adverse drug events, drug interactions, clinical decision support systems (cdss), ddi alerts

## Abstract

Introduction

Drug-drug interactions (DDIs) have the potential to harm patients. Hence, DDI alerts are meant to prevent harm; as a result, their usefulness is reduced when most alerts displayed to providers are ignored. This study aims to explore the rates and reasons for overriding alerts of DDI.

Methods

This is a retrospective study of DDI alert overrides that occurred between January 2020 and December 2020 within the inpatient medical records at a tertiary hospital, Medina City, Kingdom of Saudi Arabia.

Results

A total of 7,098 DDI alerts were generated from inpatient settings, of which 6,551(92.2%) were overridden by the physicians at the point of prescribing. “Will Monitor as Recommended” (33%) was the most common reason for the override, followed by ‘Will Adjust the Dose as Recommended (27.1%),” “The Patient Has Already Tolerated the Combination” (25.7%), and “No Overridden Reason Selected” (13.0%).

Discussion

The DDI alert overriding is still high and is comparable to other studies. However, this study reveals that physicians are ready to deal with the consequences of around 58% of DDI alerts. Additionally, 13% of physicians were not willing to report the reason for overriding. This indicates an urgent need to review and restructure the DDI alert system.

Conclusion

The DDI alert override rates are high, and this is undesirable. It is recommended to revise the DDI alert system. Future studies should dig deep for real reasons for overriding and seek inputs from all stakeholders, including developing actionable metrics to track and monitor DDI alerting system.

## Introduction

Medication errors have been highlighted as a leading cause of patient harm, with worldwide error rates ranging from 9% to 20% depending on the patient's area of care [1]. When a medication causes harm, it is called an adverse drug event (ADE). ADEs are an enormous reason for morbidity and mortality in medical institutions [2]. One important contributing factor to ADEs is drug-drug interactions (DDIs), which occur when two drugs being taken simultaneously interfere with one another's expected effects [3]. DDIs are responsible for 5%-14% of all ADEs in hospitalized patients [4]. Although there are multiple opportunities to prevent ADEs during the medication process where medications are ordered, transcribed, dispensed, and administered, it is extremely challenging for prescribers to identify potentially dangerous DDIs due to a large number of prescriptions and potential drug interaction combinations [5]. To overcome this challenge, many hospitals have implemented a computerized physician order entry system (CPOE) in combination with clinical decision support systems (CDSSs), which provides an opportunity to detect potential DDIs and alert prescribers [4]. CPOE can significantly reduce the number of prescription errors, even with limited clinical decision support (CDS) [6]. CDSSs are intended to support clinicians in their decision-making by giving them real-time, relevant, patient-specific information and guidance [7]. These systems can provide real practical benefits and significant cost savings by alerting physicians to necessary drug dosage adjustments, for example, based on the patient's renal function. Additionally, it is vital to ensure patient safety by monitoring and preventing harmful drug interactions and contraindications [8]. Medication-related CDS is an effective way to reduce errors and ADEs [9].

Although CPOE and CDSS are widely adopted in hospitals around the world, there is varying quality of evidence about their effectiveness in reducing medication errors and associated harm [10]. However, many institutions experience high override rates ranging from 46% to 98% of DDI alerts. and they are varied based on the pair of medications [4,11]. This has generated concerns about "alert fatigue," when receiving too many alerts may make providers less responsive to such warnings. Accordingly, providers face an excessive volume of relevant and irrelevant alerts, resulting in information overload, making appropriate and clinically relevant alerts more prone to be overridden. Moreover, it gives a false sense of safety with an increased risk of adverse events, as the prescribers tend to heavily rely on the alerting system [12,13]. Recently, two studies were conducted in the Saudi healthcare system revealing conflicting results; CPOE system was able to significantly reduce drug-related problems in the pediatric population (44.8% vs 35.8%) in the first study [14], while the other study found no statistically significant differences in the frequency of medication errors after introducing CDSS alerts [15]. Therefore, addressing this discrepancy necessitates conducting a study to assess and evaluate the extent of DDI alerts, override rates, and describe the reasons for overriding DDI alerts.

## Materials and methods

This is a retrospective study in which DDI alerts produced by CDSS were reviewed for the whole Georgian year of 2000. The study setting is a tertiary hospital with a total capacity of 326 beds in Al Madinah, Kingdom of Saudi Arabia. The sample included all patients admitted to the hospital for whom a DDI alert was presented to the ordering provider between the beginning of January and the end of December 2020. Alerts presented to non-prescribers (pharmacists) were excluded.

The data were collected from system-produced reports of medication orders and medication-related CDSS alerts in the inpatient settings. The investigators and two pharmacists reviewed and assessed the documented reasons for DDI alert overrides, and an 80% consensus on the proper reason was attempted and reached. As a result, the investigators determined the efficacy of CDSS alerts and the responses of the prescribing physicians and presented recommendations for the need to restructure the alerting system, if necessary.

## Results

Out of the 92,272 drug orders in the year 2000, 7,098 DDI alerts were generated from inpatient settings and 6,551(92.2%) were overridden by the physicians at the point of prescribing, as shown in Table 1.

**Table 1:** Frequency and percentage of alerts generated and overridden alert

Total prescription	Type of alerts	Total alerts (N)	Alerts overridden, N (%)
92,272	Drug-drug interaction	7,098	6,551 (92.3)

Upon reviewing the documented reasons, the investigators had to rearrange the seasons based on specific themes. After two rounds of assessing the reasons, we found that the physicians cited many different reasons for overriding the alerts; the most common reason given for overriding DDI alerts was that the physicians “will monitor the DDI reaction, as recommended” if any interaction is encountered (33.0%).

Next reason for overriding the alert and continuing the ordered medication was stated as he or she “would adjust the dose as recommended” if a reaction was encountered (27.1%). While 25.7% of alerts were overridden because the patient has already tolerated the combination before. Noticeably, that no overridden reason was selected by physicians is accounted for 13% of all override alerts. Finally, different reasons were provided as free text in 0.6% of all alerts. Table 2 shows the reasons for overriding the DDI alerts.

**Table 2:** Reasons selected upon overriding alert

Reasons for override alerts	Alerts overridden (N)	Percentage alerts overridden (%)
Will monitor as recommended	2,168	33.0
Will adjust the dose as recommended	1,781	27.1
The patient has already tolerated the combination	1,690	25.7
No reasonable alternatives	13	0.1
No overridden reason selected	854	13.0
Other (with free text reason provided)	45	0.6
Total	6,551	100

## Discussion

Computerized order entry linked with CDS holds great promise for improving medication safety, quality, and efficiency. However, many hospitals have not achieved the desired results, and DDIs have been an especially complex technology and hence impacts how physicians practice response to DDI alerts. Although the overriding rates of DDI alerts are varied from one study to another, it kept high throughout the last 20 years. Early studies found that 88%, 95%, and 58% of all alerts were overridden [16-18], while the most recent ones have almost the same rates (91%, 94%, and 96%) [19-20]. In this study, after a retrospective report review, it was found that out of 92,272 orders, a total of 7,098 DDI alerts were generated from the inpatient settings. Of all alerts, 92% were overridden throughout the study period. The results of this study bear some similarities to other studies assessing physicians’ decisions to override alerts for DDIs [17,19-21]. This can contribute to the increased number of alerts in today’s healthcare practice due to the striving to improve patient safety and adopting more sophisticated CDDSs. Although the aims of CPOE and CDSS are to reduce DDIs, it tends to generate too many clinically inappropriate alerts [17,22]. This is an important issue in informatics today, as electronic health records are now widely deployed, and almost all of them are developed by vendors. One study revealed that when the threshold for alerting was set too low, doctors overrode 89.4% of high-severity medication interaction alarms and 91.2% of drug-allergy alerts [23]. Too many alerts can lead to alert fatigue, which can cause doctors to ignore even important clinical alerts [24]. Another study reported that approximately 60% of overrides of alerts were appropriate and that the override rates varied based on the type of care setting and the chosen pair of medications [2]. As a result, we can conclude that the wide variation in the rate of overriding alerts may be due to the different definitions of DDI alerts across organizations, different levels of valuation of the alert, and finally different periods that DDI alerts were observed [4].

For the reasons of overriding the DDI alerts, our study reveals that approximately 86% of alerts were not accepted by physicians, as they stated that they can tolerate the consequences of any reactions encountered. Physicians stated that they can monitor reactions and can adjust drug doses if necessary, while 25% of physicians claimed that the patient has already tolerated the combination. Disclosing that the types of alerts were appropriately overridden reveals that duplication medication alerts were overridden 98.0% of all times, followed by drug allergy alerts (96.5%), non-formulary medications (82.5%), DDI (26.4%), age-based medication (26.4%), and alerts associated with renal insufficiency (2.2%) [2,23-25].

The current study reveals that some physicians provide no reason for overriding of all override alerts (13%). This indicates a lack of the ability to use automated systems to accommodate the reasons for prescribing combinations of drugs that could harm patients. Nevertheless, this is better than a previous study in which 53% of all overriding alerts were with no written reasons, and this can attribute to the mandatory reporting of the reason for overriding alerts [5].

Although major streams of literature prove the benefits of CDSS in enhancing patient safety and reducing medication errors, the overridden rates of alerts are still as high as 96%. Therefore, it is evident that there is a need for improving CDSS alert responsiveness and reducing alert fatigue. Furthermore, many healthcare providers overlook assessing the clinically significant of alerts. This cannot be achieved without considering the inputs from all stakeholders, including users (physicians), drugs, and institutional factors that impact the prescribing practice, and developing actionable metrics to assess the need and clinical significance of alerts and the pattern of overriding DDI alerts [4]. For reasons for overriding, more research is needed to explore the human factors elements that influence provider behavior, including non-clinical motivations of providers, such as patient demand, workload, time constraints in a busy office practice, attitudes toward disease or patients, habits, and peer influence.

Like other studies, this study has inherent limitations. First, this study was conducted in one organization, and therefore generalizing the results over the other organizations should be approached cautiously. Secondly, the impact on patient outcomes (such as adverse events) has not been investigated. Next, alert overrides and reasons for overriding alerts are not broken down by physician specialty to protect physician anonymity. Finally, physician-accepted alerts (leading to drug prescription cancellations or changes) were not detailed in the retrieved data reports and could not be analyzed beyond their frequency.

## Conclusions

This research may have shed light on medication-related alert generation and handling DDI alerts at a tertiary hospital in Medina City, Kingdom of Saudi Arabia. To improve clinical practice and manage potential drug interactions, it is essential to carefully assess the effectiveness of CDSs, prescribing procedures, and current alert systems. Potential areas for future research include investigating prescriber perceptions of generated drug-related warnings and targeting prescribers to document suggestions for improving their experience with CPOE and CDSS. Future research could also focus on ways to obtain information that helps analyze documented reasons, such as overwriting alert entries to ensure efficient use of the system.

## References

[1] Carver N, Gupta V, Hipskind JE (2023). Medical error. StatPearls [Internet].

[2] Nanji KC, Seger DL, Slight SP (2018). Medication-related clinical decision support alert overrides in inpatients. J Am Med Inform Assoc.

[3] Meslin SM, Zheng WY, Day RO, Tay EM, Baysari MT (2018). Evaluation of clinical relevance of drug-drug interaction alerts prior to implementation. Appl Clin Inform.

[4] Villa Zapata L, Subbian V, Boyce RD (2022). Overriding drug-drug interaction alerts in clinical decision support systems: a scoping review. Stud Health Technol Inform.

[5] Grizzle AJ, Mahmood MH, Ko Y (2007). Reasons provided by prescribers when overriding drug-drug interaction alerts. Am J Manage Care.

[6] Gandhi TK, Weingart SN, Borus J (2003). Adverse drug events in ambulatory care. N Engl J Med.

[7] Berner ES (2020). Clinical Decision Support Systems: State of the Art. https://digital.ahrq.gov/sites/default/files/docs/page/09-0069-EF_1.pdf.

[8] Kaushal R, Jha AK, Franz C (2006). Return on investment for a computerized physician order entry system. J Am Med Inform Assoc.

[9] Nuckols TK, Smith-Spangler C, Morton SC (2014). The effectiveness of computerized order entry at reducing preventable adverse drug events and medication errors in hospital settings: a systematic review and meta-analysis. Syst Rev.

[10] Gates PJ, Hardie RA, Raban MZ, Li L, Westbrook JI (2021). How effective are electronic medication systems in reducing medication error rates and associated harm among hospital inpatients? A systematic review and meta-analysis. J Am Med Inform Assoc.

[11] Poly TN, Islam MM, Yang HC, Li YJ (2020). Appropriateness of overridden alerts in computerized physician order entry: systematic review. JMIR Med Inform.

[12] Justinia T, Qattan W, Almenhali A, Abo-Khatwa A, Alharbi O, Alharbi T (2021). Medication errors and patient safety: evaluation of physicians' responses to medication-related alert overrides in clinical decision support systems. Acta Inform Med.

[13] Naeem A, Alwadie AF, Alshehri AM (2022). The overriding of computerized physician order entry (CPOE) drug safety alerts fired by the clinical decision support (CDS) tool: evaluation of appropriate responses and alert fatigue solutions. Cureus.

[14] AlAzmi AA, AlHamdan H, Ahmed O, Tomlin S, Rashed AN (2019). Impact of the e-prescribing system on the incidence and nature of drug-related problems in children in a Saudi hospital. Int J Pharm Pract.

[15] Khreis N, Lau AS, Al-Jedai A (2019). An evaluation of clinical decision support and use of machine learning to reduce alert fatigue. Int J Comput Commun Eng.

[16] Payne TH, Nichol WP, Hoey P, Savarino J (2002). Characteristics and override rates of order checks in a practitioner order entry system. Proc AMIA Symp.

[17] Weingart SN, Toth M, Sands DZ, Aronson MD, Davis RB, Phillips RS (2003). Physicians' decisions to override computerized drug alerts in primary care. Arch Intern Med.

[18] Shah NR, Seger AC, Seger DL (2006). Improving acceptance of computerized prescribing alerts in ambulatory care. J Am Med Inform Assoc.

[19] Wright A, McEvoy DS, Aaron S (2019). Structured override reasons for drug-drug interaction alerts in electronic health records. J Am Med Inform Assoc.

[20] Edrees H, Amato MG, Wong A, Seger DL, Bates DW (2020). High-priority drug-drug interaction clinical decision support overrides in a newly implemented commercial computerized provider order-entry system: Override appropriateness and adverse drug events. J Am Med Inform Assoc.

[21] Humphrey K, Jorina M, Harper M, Dodson B, Kim SY, Ozonoff A (2018). An investigation of drug-drug interaction alert overrides at a pediatric hospital. Hosp Pediatr.

[22] Wolfstadt JI, Gurwitz JH, Field TS, Lee M, Kalkar S, Wu W, Rochon PA (2008). The effect of computerized physician order entry with clinical decision support on the rates of adverse drug events: a systematic review. J Gen Intern Med.

[23] Lin CP, Payne TH, Nichol WP, Hoey PJ, Anderson CL, Gennari JH (2008). Evaluating clinical decision support systems: monitoring CPOE order check override rates in the Department of Veterans Affairs' Computerized Patient Record System. J Am Med Inform Assoc.

[24] Peterson JF, Bates DW (2001). Preventable medication errors: identifying and eliminating serious drug interactions. J Am Pharm Assoc.

[25] Kuperman GJ, Bobb A, Payne TH (2007). Medication-related clinical decision support in computerized provider order entry systems: a review. J Am Med Inform Assoc.

